# Sensitivity of a Dynamic Model of Air Traffic Emissions to Technological and Environmental Factors

**DOI:** 10.3390/ijerph192215406

**Published:** 2022-11-21

**Authors:** Francisco A. Buendia-Hernandez, Maria J. Ortiz Bevia, Francisco J. Alvarez-Garcia, Antonio Ruizde Elvira

**Affiliations:** Departamento de Física y Matemáticas, Universidad de Alcalá, Alcalá de Henares, 28871 Madrid, Spain

**Keywords:** air traffic emissions, dynamic system model, Morris and Sobol sensitivity analysis, dynamic control, technological innovation, environmental feedback

## Abstract

In this study, we introduce a sensitivity analysis of modelled CO${}_{2}$ aviation emissions to changes in the model parameters, which is intended as a contribution to the understanding of the atmospheric composition stabilization issue. The two variable dynamic model incorporates the effects of the technological innovations on the emissions rate, the environmental feedback, and a non-linear control term on the passengers rate. The model parameters, estimated from different air traffic sources, are subject to considerable uncertainty. The stability analysis of Monte Carlo simulations revealed that, for certain values of the non-linear term parameter and depending on the type of flight, the passengers number at some equilibrium points exceeded its initial value, while the emissions level was below the initial corresponding one. The results of two global sensitivity analyses indicated that the influence of the non-linear term prevailed on the passengers number rate, followed distantly by the environmental feedback. For the emissions rate, the non-linear term contribution dominated, with the technological term influence placing second.

## 1. Introduction

Global Sensitivity Analysis (GSA) is a term used to design a number of methodologies currently applied to the analysis of the simulations performed with complex models [1,2,3,4,5,6,7,8,9]. These encompass models whose parameters are largely unknown, models that include strong non-linearities or models with many variables. This is the case of many environmental models [10,11,12,13,14,15]. GSA applied to the analysis of their simulations can help improve the understanding of the controlling processes, to guide model development, and to target new observations to reduce parameter and prediction uncertainty. In the present study, GSA was applied to the analysis of a dynamic model of the evolution of air passengers numbers and the associated emissions ([16], from now on BH2021), under different scenarios of economic growth, technological improvements, and socioeconomic responses to the environmental problem. Aviation, one of the greatest connectors of people in the world, produces greenhouse gases (GHG) (as carbon dioxide (CO${}_{2}$), water vapour (H${}_{2}$O), nitrogen oxides (NOx), and sulphate aerosols, the remnants of incomplete combustion and particulates). These emissions produce changes in the atmospheric processes, which lead to changes in atmospheric radiation and ultimately to warming, and might also have consequences for human health [17]. However, in contrast to terrestrial modes of transport, emissions take place at different altitudes and their effects on atmospheric radiation are far more complex. Several studies estimate approximately a 5% contribution to climate change from global aviation [18]. However, unlike other means of transport, aviation occupies a preferential place as a public transport that is heavily subsidized. Therefore, the control of aviation emissions is feasible while that of other transports (for instance road transport) is more difficult. Despite the 2008 economic crisis, world air traffic and the associated emissions have continued to grow. The second IPCC report [19] already pointed out the need of a reduction in aviation emissions. The proposed measures included a systematic emissions monitoring and considered mainly technological improvements in machines (engines, carriers design) or fuels and also in aviation management procedures. The report also puts forward the assumption of the Global Warming Potential (GWP), which is still in use as a way of comparison of the effects of CO${}_{2}$ and non-CO${}_{2}$ emissions [20]. Recent studies, as Ref. [18] or [21] proposed other metrics in order to measure the effects of different GHG and how it affects the Earth radiative equilibrium.

A number of models for the evolution of air passengers and related emissions were proposed, as Ref. [18] or [22,23,24,25], which took into account the effects and issued projections for the future emissions and even the subsequent warming under different economic scenarios. The two studies Refs. [23,24], in which the 1996–2008 economic scenarios were considered, found that the introduction of the effects of technological progress could not achieve a stabilization of CO${}_{2}$ emissions in the midterm (projections for 2008–2025). Such a stabilization was obtained in Ref. [18], but only by relying on the Randers economic scenarios [26] and considering a quite optimistic estimation of the reductions introduced by the technological progress [27,28].

Recently, the stabilization of CO${}_{2}$ emissions caused by aviation has been addressed using a different approach: dynamic modelling tools (BH2021). A differential equations system that relates the evolution over time of two variables, the number of passengers per km and year (Pass/km year) and the CO${}_{2}$ mass emissions per km and year (Mton/km year), was used to study the effect that improved management and technologies or environmental strategies or some non-linear control term might have on the emissions. Although the model parameters were identified from different datasets, they presented a number of uncertainties, which were explored here using GSA methods. These would identify sections of the model parameter space leading to a similar evolution of the variables in time.

The aim of this study was the systematic analysis of the sensitivities of this dynamic system model. Details of the model are described in Section 2. The data used to identify the model parameters values together with their uncertainties and the methodology applied, in particular GSA, are described in the Section 3. The results obtained are presented and discussed in Section 4. We finish with a summary in Section 5 and some conclusions in Section 6.

## 2. Models of the CO${}_{2}$ Emissions Generated by Air Traffic

One of the model variables is x(t), the number of passengers in millions (Mpas) per km and year, a proxy for the number of flights, the other being y(t), the tons of CO${}_{2}$ emitted per Mpas and kilometre, the standard variable in the evaluation of CO${}_{2}$ emissions by transport. The evolution of both variables is linked by the relationships

(1a) $$x^{\prime }\left(t\right)=f(x(t),y(t))$$

(1b) $$y^{\prime }\left(t\right)=g(x^{\prime }\left(t\right),y\left(t\right))$$

where ’ denotes a derivative with respect to the time. For small deviations of equilibrium, given that the functions f(x,y) and g(x,y) and their first partial derivatives are continuous, the basic equations were

(2a) $$x^{\prime }\left(t\right)=a_{11}x\left(t\right)+a_{12}y\left(t\right)$$

(2b) $$y^{\prime }\left(t\right)=a_{21}x^{\prime }\left(t\right)+a_{22}y\left(t\right)$$

where $a_{12},a_{22}<0$. Notice that the first feedback terms in the right-hand side of Equations (2a) and (2b) have different mathematical structures. In the first equation, the feedback term with the $a_{11}$ represents the annual growth rate of passengers. The second term with $a_{12}$ stands for the socioeconomic response to the increased levels of Greenhouse Gases (GHG). These include the cancellations due to the increased operative costs related to the impacts of global warming (for example, increased airport fees to cover additional costs in infrastructure or administration maintenance). Moreover, the term gives way to the cancellation caused by the effect of the airline operator policies or governmental strategies for the protection of the environment. It also includes cancellations due to the passengers environmental awareness, choosing for instance alternative transport means. In the second equation, the first term on the right, (with $a_{21}$) is an empirical relationship between the change of CO${}_{2}$ emissions in mass/km year and the change of passengers number/km year. Lastly, the second term on the right hand side of this equation (with $a_{22}$) represents the reduction of the aviation emissions provided by the continuous technological improvement (in engines designs, airships, new sustainable fuels, and flight management). Many of these technological improvements originated from some programs issued by concerns about the consequences of the changes in the atmospheric composition. Therefore, this term can also be viewed as a feedback term.

These equations can be written directly in term of the variables *x*(*t*), *y*(*t*) as

(3a) $$x^{\prime }\left(t\right)=a_{11}x\left(t\right)+a_{12}y\left(t\right)$$

(3b) $$y^{\prime }\left(t\right)=a_{21}a_{11}x\left(t\right)+\left(a_{22}+a_{21}a_{12}\right)y\left(t\right)$$

The autonomous system of Equation (3) has only one equilibrium point at (0,0), a saddle point, which is unstable. Therefore, given the observed initial conditions and the parameter values considered, the variables values will grow for all the flight cases studied there (as detailed in BH2021 Appendix 2). This growth can be modified, however, by the introduction of a summand in the first feedback term of the second member of the Equation (3a), leading to Equation (4a). This term stands for the response of the variable passengers to an insecurity perception due to some crisis (climatic, volcanic, pandemic, etc.) and its effects on air transport [29]. It includes the parameter β and a dimensionalizing constant N that makes the ratio (x(t)/N) adimensional. In BH2021, N was assumed to be the value of the initial number of passengers. The second feedback term in the same equation was also modified by a factor that will reduce the negative feedback of environmental factors on the passengers number rate of change. The insecurity perception will displace the environmental awareness to a second plane. Thus, the model equations could be written as

(4a) $$x^{\prime }\left(t\right)=a_{11}\left(1-\beta \frac{x(t)}{N}\right)x\left(t\right)+\frac{a_{12}}{\left(1+\beta \frac{x(t)}{N}\right)}y\left(t\right)$$

(4b) $$y^{\prime }\left(t\right)=a_{21}a_{11}x\left(t\right)+\left(a_{22}+a_{21}a_{12}\right)y\left(t\right)$$

A summary of the definition of the coefficients can be found in Table 1. The equations are defined for negative values $a_{12}$ and $a_{22}$, the equilibrium points are spiral sinks, and the eigenvalues and eigenvectors have a complex conjugate form. More details on the characteristics of the equilibrium points and of the solutions are given in Appendix A.

## 3. Data and Methodology

### 3.1. Data

The coefficients of the linear model were assigned values according to the available data sources (detailed in the Table S1 of the Supplementary Material). The sample average annual growth rate in air traffic passengers from 2005 to 2016 was approximately 6%. This estimate could rise to 7% considering the years prior to 2005 as well (Refs. [21,30]). A number of scenarios regarding future air traffic levels from 2008 to 2025 from different predictors of the economic drivers, did forecast an increase of air traffic at 4.7%/year, as stated in Ref. [21]. However, Ref. [18] analysed simulations of the aviation sector relying on the Randers scenarios, which considered a worldwide saturation effect of economic growth, leading to a growth rate of 1.2%/year in 2050.

Data on the relationship between air traffic and emissions [31,32,33] were used to give a value to the *a*${}_{21}$ coefficient. The main information was an estimate of the mass of CO${}_{2}$ produced per passenger and kilometre, which depended on the distance travelled, allowing a classification into flight types, as detailed in Table 2. Although these values are widely accepted and used in most of the referenced studies, they are subject to uncertainties which, in the case of emissions, may be due to the approximations used in the computational models, as indicated among others by [34,35,36,37,38,39].

The range of acceptable values for the parameter a${}_{22}$, which represents the reduction of emissions due to technological innovation (in engines, fuel, or management) was obtained from Ref. [23] and also from [40,41]. According to these studies, the coefficient could range from −0.015 to −0.04. However, the recent analysis by Grewe et al. (Ref. [18]) lowered this last value to −0.05. In addition, in order to appraise the environmental feedback parameter, a${}_{12}$, two studies that took into account the higher costs related to climate change [42,43] were considered. According to these, the increase in extreme weather events will increase maintenance costs and management costs, in addition to being related to the cancellation of flights caused by convective storms, especially in autumn. Another of the studies used to estimate the effects of cancellation focused on the environmental and mental attitudes of air traffic passengers [44,45,46,47]. Since the turn of the century, when the climatic effects of CO${}_{2}$ emissions due to air traffic became public, the conflict between environmentalism and the frequent use of air flights was evident. For this reason, some airlines have offered their passengers the possibility of including a ‘CO${}_{2}$ compensation’ in their plane ticket, a possibility being used by a maximum of 1% of passengers.

However, there are movements that seek a true cancellation of the CO${}_{2}$ footprint, by diverting passengers to cleaner transport means or avoiding unnecessary trips and replacing them with telematic meetings. The values assigned to the parameter a${}_{12}$ could vary between −0.05 and −0.1. The greater absolute value was a very conservative estimate, considering 4% of the cancellation induced by increases in fuel taxes and airport charges. The cancellation rate attributed to the growing environmental awareness of passengers can range from 1% to 6%, depending on the other conditions as well. After the 2008 crisis, for example, a significant number of executives travel (estimated at 3%), were cancelled [48].

In the simulations of BH2021, the values assigned to the β parameter, which plays an essential part in the control of the solutions of the system, varied between 0.5 and 1.5. The range considered here for this parameter has been widened, from 0.1 to 3.5. On the other hand, the solutions of the system did not seem very sensitive to the different choices of the scaling constant N, which in BH2021 was assigned a value corresponding to the number of passengers/km year of the previous year. Its value in the present work was estimated, with a criterion different to the one previously used, as the number of passengers averaged to the period 1990–2015.

Further details on the data sources are given in the Table S1 of the Supplementary Materials.

### 3.2. Methodology

In the present study, the complexity of the model is characterized by the structure of its dynamic matrix, stated in its phase space diagram, which determines the asymptotic behaviour of the solutions.

A preliminary estimation of the range of variability of the β parameter was based on the short term evolution of the model solutions. The lower value of this parameter was naturally set equal to 0.1, while in order to determine the upper value, Monte Carlo numerical integrations of the model, varying that parameter value, were conducted for the three types of flights, starting from the observed initial conditions and extending for 20 years (1990–2010). The change of sign in the slope of the solutions of the emissions variable, determined from the ratio between the slope estimated from the two last years of the simulation and those obtained from the two first years, was used as an indicator in order to determine the upper value of variability of the nonlinear parameter β. In the case of the S flights, this value was fixed to include a representative number of solutions presenting a change of sign in the slope values. For the sake of comparison, the range of variability of β determined for the (S) flight was also maintained in the sensitivity studies for the other flight types. However, it was thought interesting to include a case study, for small values of the β parameter, in this research.

In order to obtain a better understanding of the importance of the different factors of the model in its response, the procedure known as Sensitivity Analysis (SA) can be very useful in order to develop, evaluate, and improve complex models [49,50,51,52]. There are two main approaches in SA: local and global [53]. In the former, individual factors are perturbed while all other factors are held fixed and variations in the output are measured. However, local sensitivity methods are unreliable for all but the simplest of models due to interactions between factors and non-linear relationships between input factor ranges and the model output [54,55]. On the contrary, in global sensitivity analysis, all factors are changed together across the full multi-dimensional input space. This approach is considered to be model independent, and the interactions between factors may be explored. Confalonieri et al. [56] list three classes of global SA techniques: regression, screening, and variance-based methods. In our case we are going to use the Morris screening method and the Sobol method based on variance.

The Morris method allows for the analysis at different points of the factor input space, and is therefore considered a global rather than a local sensitivity technique, as pointed by Refs. [54,57]. Factors ranked as important using the Morris method can be further analysed using a global sensitivity method such as Sobol. The combination of the Morris method followed by the Sobol method is an established methodology that has been successfully implemented for sensitivity analyses across a diverse range of disciplines including environmental and biological sciences, as Refs. [15,58,59].

The projections performed for the sensitivity analysis originally covered the period (1990–2014). However, they were extended for cases where it seemed necessary to complete the results, sometimes up to the year 2070.

#### 3.2.1. Morris Sensitivity Analysis

In the Morris method the input space of each factor has *p* levels in the uniform [0, 1] probability distribution function (PDF), which is rescaled for the actual value that is used in the model. The Elementary Effect ($EE_{i}$) for the ith input factor is calculated from the successive runs using

(5a) $$EE_{i}^{j}\left(x\right)=\frac{y\left(x_{1},\ldots ,x_{i}+\Delta_{j},\ldots ,x_{n}\right)-y\left(x_{1},\ldots ,x_{i},\ldots x_{n}\right)}{\Delta_{j}}$$

where $\Delta_{j}=\frac{p}{2(p-1)}$ and *p*, the grid size used for the screening, must be even. Each factor is modified once, resulting in (n+1) runs of the model. The procedure is repeated *r* times providing *r* elementary effects for each factor (*r* is referred to as the trajectory of the factor sample space). The sensitivity measures are the mean and the standard deviation of each elementary effect across all trajectories. The mean μ captures the impact that uncertainty in the factor input has on the model output, thus indicating important factors, while the standard deviation σ indicates non-linear responses to factor values and/or interactions with other factors.

(6a) $$\mu_{i}=\frac{1}{r}\sum_{j=1}^{r}EE_{i}^{j}$$

(6b) $$\sigma_{i}=\frac{1}{r}\sum_{j=1}^{r}{\left(EE_{i}^{j}-\mu_{i}\right)}^{2}$$

This measure is not appropriate in cases where the initial random distribution is non monotonic, because the effects could cancel each other out, and the index based on the mean would lose its reliability for ranking the factors. For these cases, as in the case of the present study, an alternative measure of the first order sensitivity index is the mean of the absolute values of the elementary effects.

(7) $$\mu_{i}^{*}=\frac{1}{r}\sum_{j=1}^{r}\left|EE_{i}^{j}\right|$$

As each run of the model represents a trajectory within the factor sample space, it is advisable to optimize the choice of trajectories to facilitate maximizing their spread in the input domain, prior to conducting the analysis, following Refs. [56,60]. The concept for spread is based on the sum of geometric distances between trajectory pairs *m* and *l*, given as

(8) $$d_{ml}=\sum_{i=1}^{n+1}\sum_{j=1}^{n+1}\sqrt{\sum_{k=1}^{n+1}{\left[X_{k}^{i}\left(m\right)-X_{k}^{j}\left(l\right)\right]}^{2}}\;\mathrm{for}\;m\neq l$$

where *n* is the number of input factors and $X_{k}^{j}\left(m\right)$ represents the *k*th coordinate of the *j*th point of the *m*th trajectory. Here, 1000 trajectories were created, and the Euclidean distances *d* between all the possible pairs of trajectories were calculated. The 50 that represented the highest value of *d* were used in the elementary effect method. The statistical significance was fixed to the equivalent of the 5% probability of type II error.

#### 3.2.2. Sobol Sensitivity Analysis

The method, initially developed by Sobol (Ref. [2]), was further refined in Refs. [57,61]. Given a model Y=f(X), where *Y* is the model output, and $X=(x_{1},\ldots x_{n})$ is the set of factors, the output variance *V*(*Y*) can be decomposed as

(9a) $$V(Y)=\sum_{i=1}^{n}V_{i}+\sum_{i=1}^{n}\sum_{j=i+1}^{n}V_{ij}+\ldots +V_{1..n}$$

(9b) $$V_{i}=V[E\left(Y|X_{i}\right)]$$

where *X* has been scaled between 0 and 1, to form a n-dimensional unit hyperspace. $X_{i}$ is the matrix where all factors values can vary, except for those of $x_{i}$. V(Y) is the total variance; $V_{i}$ is the partial variance of $X_{i}$ on *Y*, also known as ”main effect”, while $V_{ij}$ is the impact of $X_{i}$ and $X_{j}$ on the total variance minus their first order effect. Notice that *E* denotes the expected value. Using the variance decomposition, the first Sobol sensitivity index $S_{i}$ and the total effect Sobol sensitivity index $S_{i}^{T}$ are given by the expressions

(10a) $$S_{i}=\frac{V[E\left(Y|X_{i}\right)]}{V(Y)}$$

(10b) $$S_{i}^{T}=1-\frac{V[E\left(Y|X_{∼i}\right)]}{V(Y)}$$

where $X_{∼i}$, in opposition to $X_{i}$, denotes the matrix where all the factors values are held fixed except for those of $x_{i}$.

A Monte Carlo-based procedure using quasi-random sampling of model factors, was used to obtain the first order and total effects indices for each factor. From the analysis of these results, an heuristic criterion (Table 3) was derived [62].

In the case of the Sobol analysis, a seed value of 60,000 and a replication value of 1000, as recommended in Refs. [6,53,54], was used. Moreover, the robustness of the results was tested by comparing the results obtained for different seed and replication values.

Moreover, the results of the sensitivity indicators yielded by the two methods could not be expected to match exactly. The Morris sensitivity index $\mu_{i}^{*}$ measures the “mean absolute slope” of the output function with respect to the input variable, while the index $\sigma_{i}$ measures the variability of the slope (not absolute). On the contrary, the Sobol first order $S_{i}$ and total $S_{i}^{T}$ sensitivity indices measure the mutability of the output variable with respect to the input variable.

## 4. Results and Discussion

### 4.1. Model Configuration

A previous work (BH2021) made a first estimation of the values of the parameters of the linear dynamic model. For each type of flight, a couple of values established a range for the uncertainty of those parameters. The sensitivity of the model solutions to the parameter of the non-linear term was explored only for two representative cases. The solutions considered in the work were initialized from the totals for the number of passengers and for the emissions.

In the present study, some new uncertainty ranges, corresponding to each type of flight, were determined using recent data and research works [63,64,65,66]. As in this type of analysis the uncertainty distribution does not need to be based on the experimentally observed uncertainty in the model parameters, a convenient form of input distribution was to adopt uniform ranges based on a percentage of the nominal parameter values. The range goes from the lowest of these values, case (0), to the highest one, case (1) (Table 4). As mentioned above, each set of parameters corresponds to a different matrix, and therefore to a different dynamic.

The value of *N*, the normalization constant appearing in the non-linear term, is determined here in an alternative way to the one used previously (as the average of the passengers number to the period 1990–2015), in which the values for the different flight types were, respectively, 58.184 Mpas/year km (S), 123.642 Mpas/year km (L), and 181.826 Mpas/year km (I). Moreover, through some sensitivity experiments, described in the methodology subsection, a common range of the representative values for the insecurity parameter β, (0.1≤β≤3.5), was found.

Lastly a set of initial conditions (one per each type of flight) corresponding to the year 1990 were identified from different data bases and are presented in Table 5.

### 4.2. Characterization of the Model Complexity

The increase in the number of equilibrium points of the non-linear model with respect to those of the linear one, as result of BH2021, illustrated the complexity of the model and supported the need of the SA. In the present research, a computational scheme was used in order to obtain the solutions of the system 4a and 4b so as to perform some preliminary sensitivity experiments to changes in the β parameter of the non-linear term.

**Figure 1:**
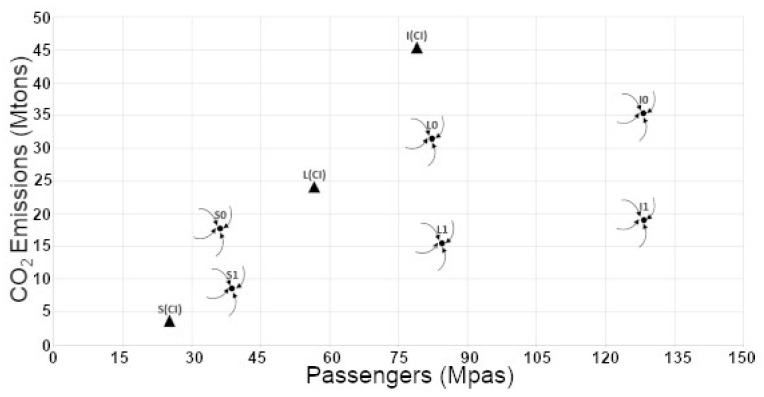
The position of the equilibrium points of the nonlinear model for β=1.25, according to the different types of flight, are marked in the phase diagram by black circles. The points with lower emission values correspond to the cases S1, L1, and I1 while those with the higher emissions correspond to the cases S0, L0, and I0. The location of the initial conditions, which are labelled S(CI), L(CI), and I(CI), are marked by black triangles.

Moreover, a stability analysis performed according to the methodology proposed in BH2021 identified one stable equilibrium point in addition to the unstable one located at the origin. This additional node spiral sink obtained for each type of flight and each of the set of values that delimited the range of uncertainty (the cases (0) and (1)) are represented in Figure 1 for a given β value, (β=1.25)). The initial states for each type of flight are also depicted in this figure. Notice that in the case of the National flights (S), the stable equilibrium points (one per case) did correspond to points with higher passengers numbers and also higher emissions coordinates values than those of the initial conditions. In the case of the Intra-European flights (L), the passengers numbers at the equilibrium points were found noticeably to the right of (greater than) those of the initial conditions, L(CI), while the corresponding emissions values were above (case L0) or below (case L1) their initial values. Additionally, in the case of the Extra-European Flights (I) the passengers coordinate of the equilibrium points were also displaced to the right of those of the initial conditions, I(CI), while the corresponding emissions values were, in both cases, considerably below the initial ones.

**Figure 2:**
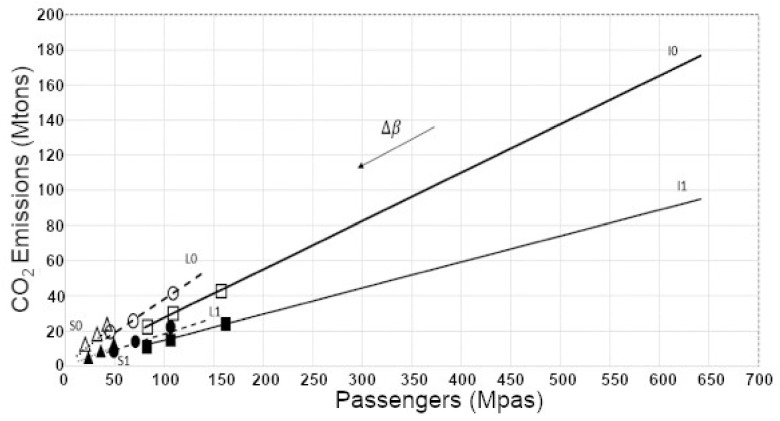
Dependence of the location of the model equilibrium points in the phase diagram, on the β values. The direction of increase of the β parameter values is indicated with an arrow. The points labelled with symbols correspond to the three cases where equilibrium points for three given β values (1, 1.5, and 2), which could be found for the three types of flights. Triangles indicate the National flights type (S), the circles depict the Intra-European flights (L), and squares correspond to Extra-European ones (I). The symbols are white when the parameter values corresponded to the case (0), while they are black in the case (1). The lines mark the position of all the other equilibrium points in the cases S (dash lines), L (dashed lines), and I (solid line). The upper lines correspond to the subcases 0 and the lower ones to the subcases 1 of each type of flight.

Furthermore, the dependence of the position of the model equilibrium points in the phase diagram on the β value was calculated and represented in Figure 2. Notice that an increase in the β parameter values is associated with a decrease of the coordinates (passengers number and CO${}_{2}$ emissions, respectively). In addition, it is evident how the equilibrium points coordinates depend on the type of flight. Both features agree with the analytical expression for the equilibrium coordinates derived in the Appendix A. For the same β value, the larger values of the passengers coordinate in equilibrium were found for Extra-European flights (I), followed by those of Intra-European flights (L), with the National flights (S) in the last place.

### 4.3. Results of the Global Sensitivity Analysis

#### 4.3.1. The GSA for a Wide Range of Non-Linear Parameter Values

Both GSA methods, Morris and Sobol, were applied to the analysis of the model simulations performed for the different types of flight. For the sake of comparison, the β parameter was allowed to vary here in the range (0.1≤β≤3.5).

**Figure 3:**
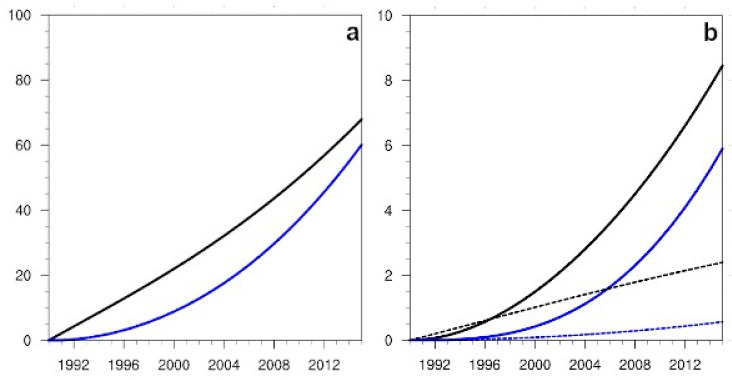
(**a**) Projections in time from the 1990 initial conditions of the Morris sensitivity indices, $\mu^{*}$ (represented with black lines) and σ (depicted with blue lines) for the variable passengers, in the case of National flights (S). In order to represent both indices in the same figure, the σ index has been rescaled by a factor 10. The sensitivities to the β parameter term are depicted with solid lines, and those to the $a_{22}$ term, with short dashed lines. (**b**) The evolution over time of the Morris sensitivity parameters for the variable Emissions. Colour codes and line styles are the same as in (**a**).

The statistical indicators resulting from the application of the Morris method, $\mu^{*}$ and σ, depended on time (Figure 3a). In the case of (S) flights, and for the variable passengers (Figure 3a), the sensitivity of these indices to the non-linear term was the only relevant one, the evolution in time being linear in the first index, and non-linear in the second one. For the emissions variable (Figure 3b), differences in the sensitivities to the non-linear term appeared as differences in growth rates. The sensitivities to the other terms, such as the technological one (which includes $a_{22}$), always remained small, although the first index for this term was above the corresponding one for the nonlinear term during the first 6 years of the projection.

**Figure 4:**
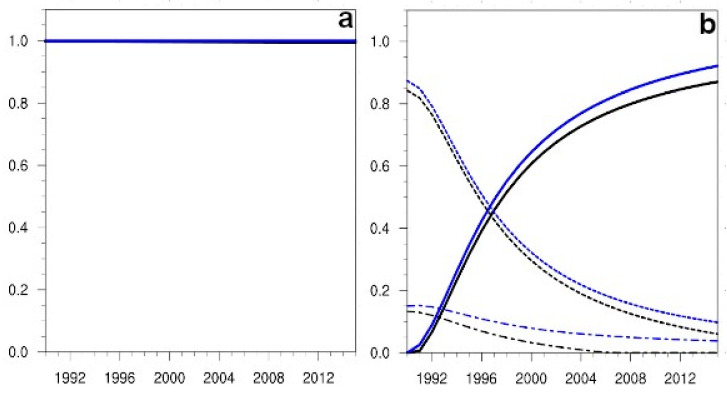
(**a**) Projection in time from the 1990 initial conditions of the Sobol sensitivity indices, first order $S_{i}$ (represented with black lines) and total $S_{i}^{T}$ (depicted with blue lines), for the variable passengers, in the case of National flights (S). Line styles are the same as in Figure 3a. (**b**) The evolution in time of the Sobol indices, first order and total, for the variable emissions. The sensitivities to the β parameter term are depicted with solid lines, those to the $a_{22}$ term with short dashed lines, and the ones to the $a_{12}$ term with short and long dashed lines.

The diagnostic of the Sobol indices had an easier interpretation thanks to the heuristic criterion presented in Table 3. Thus, for the same flight type (S), in the case of the variable passengers, the sensitivity to the non-linear term of both indices (S${}_{i}$ and S${}_{i}$${}^{T}$) maintained a constant and very relevant value during all the simulations (Figure 4a). For the emissions variable, both indices highlighted the relevance of the innovation term in the first year of the simulation, while the sensitivities to the non-linear term became relevant only after some years (6 or 7 depending on the index) (Figure 4b), a feature also detected by the Morris indicators. The sensitivities to the cancellation term remained in the irrelevant sector (below 0.3) for the whole simulation length. However, as the distance between the two Sobol indices of the cancellation term supported the importance of the interactions of this term with others, these sensitivities are depicted in all the figures for the Sobol indices when their values could be distinguish from null.

**Figure 5:**
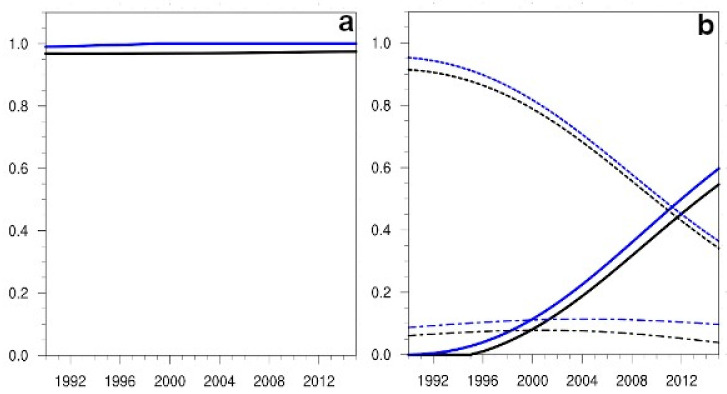
(**a**) Projection in time of the Sobol sensitivity indices, $S_{i}$ and ${S_{i}}^{T}$, for the variable passengers, in the case of Intra-European flights (L) with β values in the interval (0.1, 3.5). Colour codes and line styles are the same as in Figure 4a. (**b**) The evolution in time of the Sobol sensitivity indices, $S_{i}$ and $S_{i}^{T}$, for the emissions variable with the colour codes and line styles of Figure 4b.

The Sobol indices for the variable passengers in the (L) flights case presented similitudes with the corresponding (S) flights, concerning the relevance of the non-linear term, and the irrelevance of the cancellation one (Figure 5a). On the contrary, in the sensitivity indices for the emissions variable (Figure 5b) the innovation term dominated the first years, remaining relevant till year 20 of the projection while the indices for the non-linear term reached the relevance threshold value only in the last year. The statistical sensitivity diagnostics for the (I) flights case (not shown) were qualitatively similar to those obtained for the (L) flights case. The non-linear term was the only relevant influence for the variable passengers while the technological innovation factor was the relevant one for the variable emissions.

Additionally, the distance between the first and second sensitivity indices (Morris or Sobol) for a certain factor gives an estimation of the non-linear interaction among this and the other factors. The results showed that this interaction depended on the type of flight and through it on the β parameter interval of variability. The interaction was almost unnoticeable in the (S) flight case, and, for the same type of flight, was more noticeable in the cancellation term (corresponding to the a${}_{12}$ parameter) than in the others, as for instance the non-linear or the technological ones.

#### 4.3.2. A Case Study: The GSA for Small Values of the Nonlinear Parameter

Lastly, the analysis was focused on some case studies, where the values of the parameter of the non-linear term remained small, its range of variability being limited to the interval (0.1≤β≤0.5). In this case study, and in order to complete the results, the projections were extended up to the year 2070.

**Figure 6:**
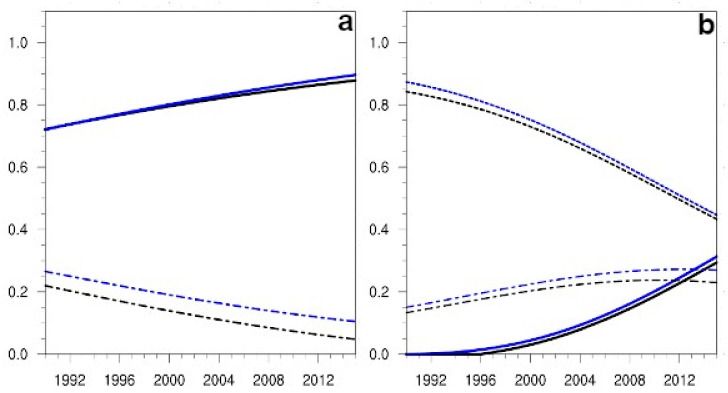
(**a**) Projection in time of the Sobol sensitivity indices, $S_{i}$ and $S_{i}^{T}$, for the variable passengers and the National flights (S), in the case study selected (β values in the interval (0.1, 0.5). Line styles and colour codes as in Figure 4a. (**b**) The evolution in time of the Sobol sensitivity indices for the emissions variable for the same flight type and case study, with colour codes and line styles as in Figure 4b.

In the (S) flights case and for the variable passengers, the Sobol indices indicated, as in the general case, a relevant sensitivity to the non-linear term, which only after the year 2000 could be considered very relevant, while those to all the other terms remained irrelevant (Figure 6a). In the case of the emissions variable, the sensitivity to the innovation term remained relevant for more than 20 years (Figure 6b) while those to the non-linear term, although increasing with time, did not reach the relevance threshold (in the case of the $S_{i}$ index) till the year 2014 and became very relevant from 2028 on. The sensitivity to the cancellation term remained in the irrelevant sector, although its values were higher than those obtained when considering the wide β value.

**Figure 7:**
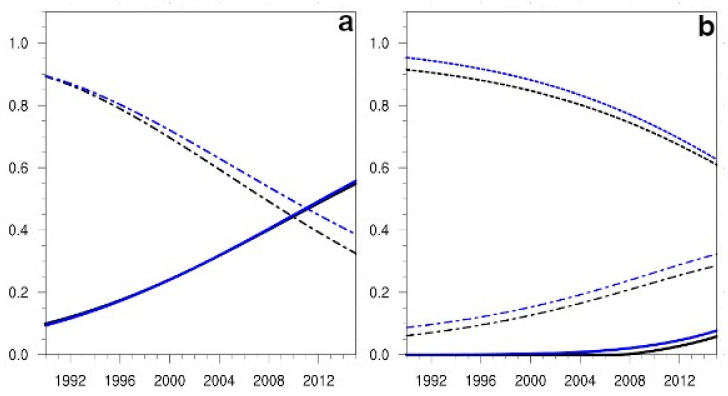
(**a**) Projection in time of the Sobol sensitivity indices, $S_{i}$ and $S_{i}^{T}$, for the variable passengers and the Intra-European flights (L) case, β values as in Figure 6a. Linestyles and colour codes are the same as in Figure 4b. (**b**) The evolution in time of the Sobol sensitivity indices for the emissions variable for the same flight type and case study, with colour codes and line styles as in (**a**).

In the case of the (L) flights and for the variable passengers, the sensitivity to the cancellation term, although decaying in time, was above the relevance threshold for 18 years, while the sensitivity to the non-linear term reached that threshold only in the last years of the simulation (Figure 7a). In the case of the emissions variable, the sensitivity to the innovation term, although decaying with time, remained relevant for the period represented (Figure 7b), while the one to the non-linear term, although growing with time, did not reach the relevance threshold till the year 2037 and became very relevant only after 2060. Thus, for the two flight types studied, the Sobol sensitivity indices found in the (L) flights behaved quite differently to those found in the (S) flights for the passengers variable, and rather similarly for the emissions variable.

## 5. Summary

Different sensitivity methods have been applied here to the analysis of a two variable dynamic model for the effect of air transport passengers on CO${}_{2}$ emissions. Although the model has a reduced number of variables and parameters, the complexity introduced by the non-linear term justified the use of GSA. The dynamic model was based on an empirical relationship between its two variables—passengers number and CO${}_{2}$ emissions—both of them per km and year. This relationship had been substantiated by the data registered by the European organizations for aviation, and its numerical value is quite different depending on the flight type. Therefore, this is the key parameter for the model solutions, which are obtained separately and using specific initial conditions according to the flight type. Then, the second relevant parameter was the non-dimensional one (β) included in the non-linear terms. The inclusion of this term was necessary in order to obtain stable equilibrium points in the solutions of the model. Although the model was proposed and justified in a previous study, its solutions were considered only for a few chosen parameter values. Here the sensitivity of the model to changes in its parameter values was consistently explored.

## 6. Conclusions

An analytical expression derived for the equilibrium points coordinates showed that these are inversely proportional to the non-dimensional (β) parameter value, included in the non-linear control term.

The range of the β parameter was determined with the help of preliminary Monte Carlo stability analysis, which yielded some novel results. For the same value of the non-dimensional parameter in the non-linear term and depending on the type of flight, equilibrium points could be located at passengers number below, near, or above their initial value while the corresponding CO${}_{2}$ emissions level was located above, near, or below its initial one. For instance, in one of the cases of the (I) flight, the passengers coordinate of the equilibrium point did sit above the initial condition while the emissions coordinate fell below the corresponding initial one. Finally, a wide range of variability for the β parameter, common to all the flight types, was determined. Additionally, a case study, where the non-linear parameter values were kept small, was also considered. Similarly, different ranges of variability for the other parameters were also identified, from data bases or relevant published studies. Appropriate parameter distributions, an important step in GSA method applications, were also selected.

Two GSA methods were applied for the characterization of the model sensitivities to changes in its parameters values. The results of both GSA showed that the sensitivity to the non-linear control term was dominant in the passengers variable while in the emissions variable the sensitivity to the technological term was also relevant, specially in the first years of the projection. It was found also that the sensitivity to the non-linear term was more important in the case of the (S) flights than in the other two cases, where the technological term was more important. The sensitivities to the cancellation term were found to be irrelevant even in the case study, when the values of the β parameter were kept small. However its interactions with those of other terms could be important.

In general, the Morris indices presented a strong dependence on time. In order to find which of the sensitivities dominated, it was necessary to compare the slopes of the different indices. That is, in the Morris analysis, the results are qualitative. On the contrary, the dependence on time of the Sobol indices, if any, was less marked than the one found in the Morris indices for the same type of flight. Moreover, the Sobol indices are normalized, its interpretation being straightforward thanks to a heuristic criterion that allows for a quantitative approach.

As the results highlight, the obtained returns of the technological investment would be greater if the technological innovation efforts were focused on the long-distance flights. The lack of sensitivity of national flights to the technological improvements points to the interest of diverting these trips to alternative transport means.

## Figures and Tables

**Table 1:** Model parameter definition.

Parameter	Definition
*a* ${}_{11}$	Feedback parameter of the number of passengers/(km year) at time t on the number of passengers/(km year) rate of change. It was obtained from the ICAO air traffic data base.
*a* ${}_{12}$	Feedback (cancellation) parameter of the CO${}_{2}$ emissions/(km year) at time t, on the passengers number/(km year) rate of change, associated to the socioeconomic response (environmental consciousness, environmental taxes, and others).
*a* ${}_{21}$	Parameter that relates the number of passengers to the CO${}_{2}$ emissions. It depends on the type of flight and can be found in the LIPASTO data base.
*a* ${}_{22}$	Parameter representing the effects of technological improvements on the CO${}_{2}$ emissions/(km year) rate of change, here called the ’technological innovation parameter’.
β	Feedback parameter of the passengers number/(km year) at time t on the passengers number/(km year) rate of change associated to a perception of insecurity (or need of control).
*N*	Dimensional constant that here is assumed as the average estimate of the passengers number/(km year) for the period analysed.

**Table 2:** Definition of the type of flight.

Case	Name	Distance	CO${}_{2}$ Production g/Passenger-km
S	National	less than 500 km	259
L	Intra-European	less than 2500 km	178
I	Extra-European	less than 5000 km	114

**Table 3:** Relevance of the Sobol indices.

Irrelevant	Little Relevant	Relevant	Very Relevant
$0\;\leq \;S_{I}\;\leq \;S_{I}^{T}<0.3$	$0.3\;\leq \;S_{I}\;\leq \;S_{I}^{T}<0.5$	$0.5\;\leq \;S_{I}\;\leq \;S_{I}^{T}<0.8$	$0.8\;\leq \;S_{I}\;\leq \;S_{I}^{T}<1.0$

**Table 4:** Dynamical matrix coefficients used in the different case studies. The *a*${}_{21}$ parameter is determined by the flight type. For each of these, two values of the cancellation parameter *a*${}_{12}$ are considered.

Case	$a_{11}$	$a_{12}$	$a_{21}$	$a_{22}$
S0	0.062	−0.05	0.259	−0.02
S1	0.064	−0.10	0.262	−0.05
L0	0.062	−0.05	0.178	−0.02
L1	0.064	−0.10	0.200	−0.05
I0	0.062	−0.05	0.114	−0.02
I1	0.064	−0.10	0.150	−0.05

**Table 5:** Initial conditions (1990) for passengers and CO${}_{2}$ emissions.

	National	Intra-European	Extra-European
MPas (CI 1990)	25.263	53.683	78.946
MTCO${}_{2}$ (CI 1990)	3.27	23.89	45.00

## Data Availability

The data used in this study are public and available at the web pages detailed in the table of the Supplementary Materials.

## Supplementary Materials

The following supporting information can be downloaded at: https://www.mdpi.com/article/10.3390/ijerph192215406/s1, Table S1: Data Sources.

## Appendix A. The Non-Linear Model

The equilibrium points of Equation (4) can be found by imposing the conditions $x^{\prime }\left(t\right)=0$; $y^{\prime }\left(t\right)=0$. From the second of these, it can be obtained

(A1) $$y\left(t\right)=\frac{-a_{21}a_{11}}{a_{12}a_{21}+a_{22}}x\left(t\right)$$

Replacing this expression in Equation (4a), we will obtain

(A2) $$x^{\prime }\left(t\right)=a_{11}x\left(t\right)\left(1-\beta \frac{x(t)}{N}\right)-\frac{a_{12}a_{21}a_{11}x\left(t\right)}{\left(1+\beta \frac{x(t)}{N}\right)\left(a_{12}a_{21}+a_{22}\right)}$$

which, under the equilibrium condition $x^{\prime }\left(t\right)=0$ will yield, for the equilibrium points, the relationships

(A3a) $$x(t)=\frac{N}{\beta }\sqrt{1-\frac{a_{12}a_{21}}{a_{12}a_{21}+a_{22}}}$$

(A3b) $$y(t)=-\frac{a_{11}a_{21}}{a_{12}a_{21}+a_{22}}\frac{N}{\beta }\sqrt{\frac{a_{22}}{a_{12}a_{21}+a_{22}}}$$

Notice that the expression under the root is positive, as the parameters $a_{12}$ and $a_{22}$ are negative by definition while the parameters $a_{11}$ and $a_{12}$ were defined as positive. Therefore, the expressions $a_{12}a_{21}$ and $a_{12}a_{21}+a_{22}$ are negative and the ratio $\frac{a_{12}a_{21}}{a_{12}a_{21}+a_{22}}$ is positive and less than one for the values used in the present work. Then, the values of the coordinates of the equilibrium points of the solution with physical meaning were found to be positive.

This way, we have derived an analytical expression for the location of the equilibrium points as a non-linear function of the model parameters, in particular of the parameter of control, β.
