# Supplementary material for: Sensitivity of a Dynamic Model of Air Traffic Emissions to Technological and Environmental Factors
Source: Int J Environ Res Public Health. 2022 Nov 21;19(22):15406. doi: 10.3390/ijerph192215406 (PMC9697098; doi:10.3390/ijerph192215406)
Supplement: Supplementary file 1 [file ijerph-19-15406-s001.zip › ijerph-2007932-supplementary.pdf]

Table S1: Data sources.

Finland Transport Research Centre (LIPASTO)	Average passenger aircraft emission and energy consumption by passenger kilometre https://www.icao.int/environmental-protection/Pages/env2016.asp
EUROSTAT	Air Transport by passengers by country (yearly data) https://ec.europa.eu/eurostat/web/transport/data/main-tables
The International Council on Clean Transportation (ICCT)	Country-specific operations and CO2 emissions data for commercial passenger transport, 2018. Supplemental data from ICCT Working Paper 2019-16 https://theicct.org/publications/co2-emissions-commercial-aviation-2018
Intergovernmental Panel on Climate Change (IPCC 1995a)	First approach at CO_2 equivalent https://www.ipcc.ch/site/assets/uploads/2018/02/ipcc_wg3_ar5_annex-ii.pdf
Intergovernmental Panel on Climate Change (IPCC 2000)	Data update of the CO_2 equivalent https://www.ipcc.ch/site/assets/uploads/2018/03/emissions_scenarios-1
Intergovernmental Panel on Climate Change (IPCC 2007)	The Role of Aircraft in Climate Change-Evaluation of Sample Scenarios (Fig. 6-15: Bar charts of radiative forcing in 2050) https://archive.ipcc.ch/ipccreports/sres/aviation/ index.php?idp=83
Intergovernmental Panel on Climate Change (IPCC 2014)	Climate Change 2014 Synthesis Report Summary for Policymakers (Fig. 4.1 Sectorial CO2 emissions by sector ant total non-CO2 greenhouse gas (GHG) emission) SPM 2.1 Key drivers of future climate Cumulative emissions of CO2 largely determine global mean surface warming by the late 21st. century and beyond. Projections of greenhouse gas emissions vary over a wide range, depending on both socioeconomic development and climate policy. https://www.ipcc.ch/site/assets/uploads/2018/02/AR5_SYR_FINAL_SPM.pdf
European Commission, Directorate-General for Mobility and Transport	Taxes in the field of aviation and their impact : final report, Publications Office, 2019, https://data.europa.eu/doi/10.2832/913591
